# Lipoproteins and metabolites in diagnosing and predicting Alzheimer’s disease using machine learning

**DOI:** 10.1186/s12944-024-02141-w

**Published:** 2024-05-21

**Authors:** Fenglin Wang, Aimin Wang, Yiming Huang, Wenfeng Gao, Yaqi Xu, Wenjing Zhang, Guiya Guo, Wangchen Song, Yujia Kong, Qinghua Wang, Suzhen Wang, Fuyan Shi

**Affiliations:** 1 Department of Health Statistics, School of Public Health, Shandong Second Medical University, Weifang, Shandong 261053 China; 2 Department of Rheumatology and Immunology, Affiliated Hospital of Shandong Second Medical University, Weifang, Shandong 261031 China

**Keywords:** Alzheimer’s disease, Random forest, Lasso regression, CatBoost algorithm

## Abstract

**Background:**

Alzheimer’s disease (AD) is a chronic neurodegenerative disorder that poses a substantial economic burden. The Random forest algorithm is effective in predicting AD; however, the key factors influencing AD onset remain unclear. This study aimed to analyze the key lipoprotein and metabolite factors influencing AD onset using machine-learning methods. It provides new insights for researchers and medical personnel to understand AD and provides a reference for the early diagnosis, treatment, and early prevention of AD.

**Methods:**

A total of 603 participants, including controls and patients with AD with complete lipoprotein and metabolite data from the Alzheimer’s disease Neuroimaging Initiative (ADNI) database between 2005 and 2016, were enrolled. Random forest, Lasso regression, and CatBoost algorithms were employed to rank and filter 213 lipoprotein and metabolite variables. Variables with consistently high importance rankings from any two methods were incorporated into the models. Finally, the variables selected from the three methods, with the participants’ age, sex, and marital status, were used to construct a random forest predictive model.

**Results:**

Fourteen lipoprotein and metabolite variables were screened using the three methods, and 17 variables were included in the AD prediction model based on age, sex, and marital status of the participants. The optimal random forest modeling was constructed with “mtry” set to 3 and “ntree” set to 300. The model exhibited an accuracy of 71.01%, a sensitivity of 79.59%, a specificity of 65.28%, and an AUC (95%CI) of 0.724 (0.645–0.804). When Mean Decrease Accuracy and Gini were used to rank the proteins, age, phospholipids to total lipids ratio in intermediate-density lipoproteins (IDL_PL_PCT), and creatinine were among the top five variables.

**Conclusions:**

Age, IDL_PL_PCT, and creatinine levels play crucial roles in AD onset. Regular monitoring of lipoproteins and their metabolites in older individuals is significant for early AD diagnosis and prevention.

## Background

Alzheimer’s disease (AD) is a chronic neurodegenerative disorder and a protein-conformational disease that is primarily caused by abnormal processing and aggregation of normally soluble proteins [1–3]. In the brains of patients with AD, β-amyloid proteins (Aβ) aggregated into plaques, and tau proteins aggregated abnormally within neurons [4–6]. The presence of these aggregates not only disrupts the normal functioning of neurons but also leads to cell death and brain tissue degeneration [7–9]. The pathological changes in AD are closely associated with the imbalanced metabolism of Aβ, leading to the formation of senile plaques, as well as the excessive phosphorylation of tau protein, which results in the formation of neurofibrillary tangles in neurons [10–12].

Some studies have reported metabolic disturbances in patients with AD, suggesting a close association between AD onset and metabolic imbalance [13, 14]. Normal lipid metabolism is crucial for maintaining the proper functioning of the brain [15–17]. Lipid metabolism disorders can lead to synaptic loss and, ultimately, memory impairment through pathways such as inflammatory response, oxidative stress, blood–brain barrier damage, mitochondrial dysfunction, and neuronal signaling pathway damage [18]. For example, cholesterol is a major component of cell membranes and myelin sheaths, playing a crucial role in maintaining synaptic integrity and neuronal function. Amyloid precursor protein (APP) trafficking, proteolytic cleavage, and Aβ aggregation related to the core pathological process of AD are all related to biofilms and are affected by membrane components [19, 20]. Whether it is increased cholesterol levels, ApoE proliferation, trace amounts of sodium sulfate, or decreased plasmalogens, they all contribute to dysfunction in Aβ processing, ultimately leading to AD. Even the slightest changes in lipid concentrations can have a significant impact on the progression and severity of AD [20]. Therefore, lipid distribution and metabolism may affect the pathogenesis of AD. Moreover, cleavage products of APP accumulate at mitochondria-associated endoplasmic reticulum membranes (MAMs), where it impairs mitochondrial bioenergetics, disrupts cellular lipid homeostasis, and leads to alterations in membrane lipid components common in AD pathogenesis [21, 22].

Lipoproteins are globular particles composed of a hydrophobic core rich in sterol lipids and triglycerides and an outer shell composed of proteins, phospholipids, cholesterol, etc. Lipoproteins can be divided into chylomicrons (CM), very-low-density lipoproteins (VLDL), intermediate-density lipoproteins (IDL), low-density lipoproteins (LDL), and high-density lipoproteins (HDL). Metabolites are small molecules of substrates, intermediates, and products in cellular metabolic processes. Abnormal lipid metabolism may lead to excessive accumulation of Aβ and abnormal phosphorylation of tau protein [23, 24]. This has a certain suggestive effect on the prediction of AD. Researchers have explored the study of Aβ and tau proteins, making significant contributions to early disease diagnosis and monitoring disease progression. However, the exact mechanisms underlying AD onset remain unclear [25], and the increasing prevalence of AD poses a substantial economic burden on nations and affected families [26].

Random forest is effective in predicting AD [27, 28]. By integrating the predictions of multiple decision trees, model accuracy can be enhanced, making random forests suitable for complex classification problems [29, 30]. Moreover, random forests are highly resilient against noise and anomalies [31], making them capable of effectively handling complex real-world data. We hypothesize that machine learning methods such as random forests can effectively screen out important variables from high-dimensional data, and some key lipoproteins and metabolites may play an important role in the development of AD. Therefore, this study aimed to use various machine learning methods to screen risk factors for AD and analyze key lipid proteins and metabolite factors that influence the onset of AD using a random forest model.

## Methods

### Data sources

Data collected from the Alzheimer’s Disease Neuroimaging Initiative (ADNI) database (https://adni.loni.usc.edu/) were used to assist in the early diagnosis and tracking of AD. Based on the status of the participants and availability of lipoprotein and metabolite data, 603 participants were recruited between 2005 and 2016, including 294 controls and 309 patients with AD. To achieve the research objectives, the lipoprotein and metabolite data of the participants were filtered, excluding variables with missing values. Ultimately, 213 lipoprotein and metabolite variables were included in this study. All 603 participants in this study had complete records for the 213 lipoprotein and metabolite variables.

### Variable screen and predictive model construction methods

In this study, three machine learning methods, random forest, CatBoost algorithm, and Lasso regression, were used to screen key lipoproteins and metabolites. First, the 213 variables were sorted and filtered based on their importance, with any variables (lipoproteins and metabolites) ranking high in importance in ≥ 2 machine learning methods included in the model. Subsequently, the selected variables, along with the age, sex, and marital status of the participants, were used to construct a random forest predictive model to identify the key factors influencing AD onset. The random forest is trained by randomly selecting a subset of features, and only some features in each decision tree participate in the partitioning process, which effectively reduces the correlation between features and provides a more reliable feature importance ranking. The CatBoost algorithm can automatically discover and utilize the interaction between features in the process of building a decision tree, better capture the nonlinear relationship between features, and improve the expressive ability of the model.

In this study, while using the Lasso regression to screen variables, the original data were first standardized with Z-scores to ensure that each feature in the model was properly standardized, thereby improving the stability of the model and reliability of the results. The result of standardization was such that the mean of each variable was 0, and the standard deviation was 1. However, the random forest and CatBoost algorithms are tree model-based algorithms unaffected by the feature scale; therefore, no standardization was applied to the data, and the analysis was conducted using raw data.

The categorical variables used in the stochastic forest prediction model were assigned different values according to their attributes. Sex was coded 1 for males and 0 for females. Marital status was coded 1 for married individuals and 0 for those who were unmarried, widowed, or divorced. Apart from sex and marital status, the other variables were continuous. The main variables and their abbreviations are presented in Table 1.

**Table 1:** Names and abbreviations of the main variables in this study

Variable	Abbreviation	Variable	Abbreviation
Acetate	ACETATE	Phospholipids to total lipids ratio in medium HDL	M_HDL_PL_PCT
Acetoacetate	ACETOACETATE	Cholesteryl esters to total lipids ratio in medium VLDL	M_VLDL_CE_PCT
Acetone	ACETONE	Omega-3 fatty acids	OMEGA_3
Albumin	ALBUMIN	Pyruvate	PYRUVATE
Apolipoprotein A1	APOA1	Cholesterol to total lipids ratio in small HDL	S_HDL_C_PCT
Citrate	CITRATE	Cholesterol esters in small HDL	S_HDL_CE
Creatinine	CREATININE	Free cholesterol in small HDL	S_HDL_FC
Glucose	GLUCOSE	Free cholesterol to total lipids ratio in small HDL	S_HDL_FC_PCT
Glycoprotein acetyls	GLYCA	Phospholipids in small HDL	S_HDL_PL
Average diameter for HDL particles	HDL_SIZE	Phospholipids to total lipids ratio in small HDL	S_HDL_PL_PCT
Cholesteryl esters to total lipids ratio in IDL	IDL_CE_PCT	Average diameter for VLDL particles	VLDL_SIZE
Phospholipids to total lipids ratio in IDL	IDL_PL_PCT	Triglycerides in VLDL	VLDL_TG
Triglycerides in IDL	IDL_TG	Cholesterol to total lipids ratio in very large HDL	XL_HDL_C_PCT
Cholesterol to total lipids ratio in large HDL	L_HDL_C_PCT	Free cholesterol to total lipids ratio in very large HDL	XL_HDL_FC_PCT
Cholesteryl esters to total lipids ratio in large HDL	L_HDL_CE_PCT	Total lipids in very large HDL	XL_HDL_L
Phospholipids to total lipids ratio in large HDL	L_HDL_PL_PCT	Phospholipids to total lipids ratio in very large HDL	XL_HDL_PL_PCT
Triglycerides in large HDL	L_HDL_TG	Triglycerides to total lipids ratio in very large HDL	XL_HDL_TG_PCT
Cholesteryl esters in large LDL	L_LDL_CE	Concentration of very large VLDL particles	XL_VLDL_P
Cholesteryl esters to total lipids ratio in large LDL	L_LDL_CE_PCT	Phospholipids to total lipids ratio in very large VLDL	XL_VLDL_PL_PCT
Phospholipids to total lipids ratio in large LDL	L_LDL_PL_PCT	Triglycerides in very large VLDL	XL_VLDL_TG
Free cholesterol to total lipids ratio in large VLDL	L_VLDL_FC_PCT	Free cholesterol in chylomicrons and extremely large VLDL	XXL_VLDL_FC
Total lipids in large VLDL	L_VLDL_L	Phospholipids in chylomicrons and extremely large VLDL	XXL_VLDL_PL
Phospholipids in large VLDL	L_VLDL_PL	Age	AGE
Triglycerides in large VLDL	L_VLDL_TG	Gender	GENDER
Average diameter for LDL particles	LDL_SIZE	Marital status	PTMARRY

In this study, a non-replacement random sampling method was used to build the random forest predictive model. The research cohort was divided into training and testing datasets in an 8:2 ratio. The training set was used to establish the random forest model, and the testing set was used to evaluate the model’s performance. Optimizing model parameters aimed to enhance the model’s ability to capture the complexity of the data [32], which was achieved by calculating the optimal number of features “mtry” and decision trees “ntree” used in each tree of the model [33]. Out-of-bag error is an estimate of the model’s performance on unused data by using out-of-bag data. The mean error rate based on out-of-bag error is an important metric used to evaluate the performance of classification models. It indicates the proportion of errors in the prediction process, and a lower mean error rate usually indicates that the model has higher accuracy and generalization performance. In this study, the minimum mean error rate of the model was calculated to determine the optimal number of feature “mtry” used by each tree in a random forest. Then, by plotting the relationship between the model error and the number of decision trees, the optimal number of decision trees used by the model is determined. Finally, utilizing the two optimal parameters, along with the settings of importance = true and num_class = 2, a random forest model is constructed.

### Statistical analysis

In this study, SPSS 21.0 (IBM Corp., Armonk, NY, USA) software was used to compare the differences in basic information between the NC group and the AD group by using two independent samples t-test or chi-square test or Mann–Whitney test, and the test level was α = 0.05. The random forest package in R version 4.2.2 was used to construct the random forest model, glmnet package for Lasso regression, and CatBoost package for the CatBoost model.

## Results

### Basic information about the participants

A total of 603 participants were recruited for this study: 294 controls and 309 in the group with AD. The average age of the controls was (74.72 ± 5.93 years), consisting of 136 males (46.26%) and 158 females (53.74%). Among them, 201 (68.37%) were married, and 93 (31.63%) were unmarried. The mean age of the group with AD was (74.34 ± 7.68 years), including 175 males (56.63%) and 134 females (43.37%). In the group with AD, 260 individuals (84.14%) were married, and 49 (15.86%) were unmarried. There was no statistically significant age difference between the two groups, but there were statistically significant differences in gender and marital status. Since this study mainly aimed to explore the key lipoproteins and metabolites that affect the incidence of AD, only the three basic variables of age, gender, and marital status were included in the final modeling, and the three variables of ethnicity, education scores, and MMSE were not included in the study. The basic information of the two groups is shown in Table 2.

**Table 2:** Baseline data of the NC group and AD group

Index	NC group (*n* = 294)	AD group (*n* = 309)	χ^2^/t/Z	*P*
Age	74.72 ± 5.93	74.34 ± 7.68	0.681	0.496
Sex				
Male (%)	136 (46.26)	175 (56.63)	6.494	0.011
Female (%)	158 (53.74)	134 (43.37)		
Marital status				
Married (%)	201 (68.37)	260 (84.14)	20.825	0.000
Unmarried, etc.(%)	93 (31.63)	49 (15.86)		
Ethnicity				
Hispanic / Latino (%)	8 (2.72)	7 (2.27)	0.129	0.719
Not Hispanic / Latino (%)	286 (97.28)	302 (97.73)		
Education scores	16.38 ± 2.71	15.41 ± 2.94	4.226	0.000
MMSE				
M(QR)	1	3	-18.669^a^	0.000

^a^Mann–Whitney test

### Variable screening results based on the random forest method

In the random forest model, the Mean Decrease Accuracy (MDA) and Mean Decrease Gini (MDG) are crucial indicators for assessing variable importance. The MDA evaluates the contribution of each variable to the model accuracy, whereas the MDG measures the improvement in Gini impurities during the decision-tree splitting process. Higher values of these indicators correspond to more significant variables.

**Fig. 1:**
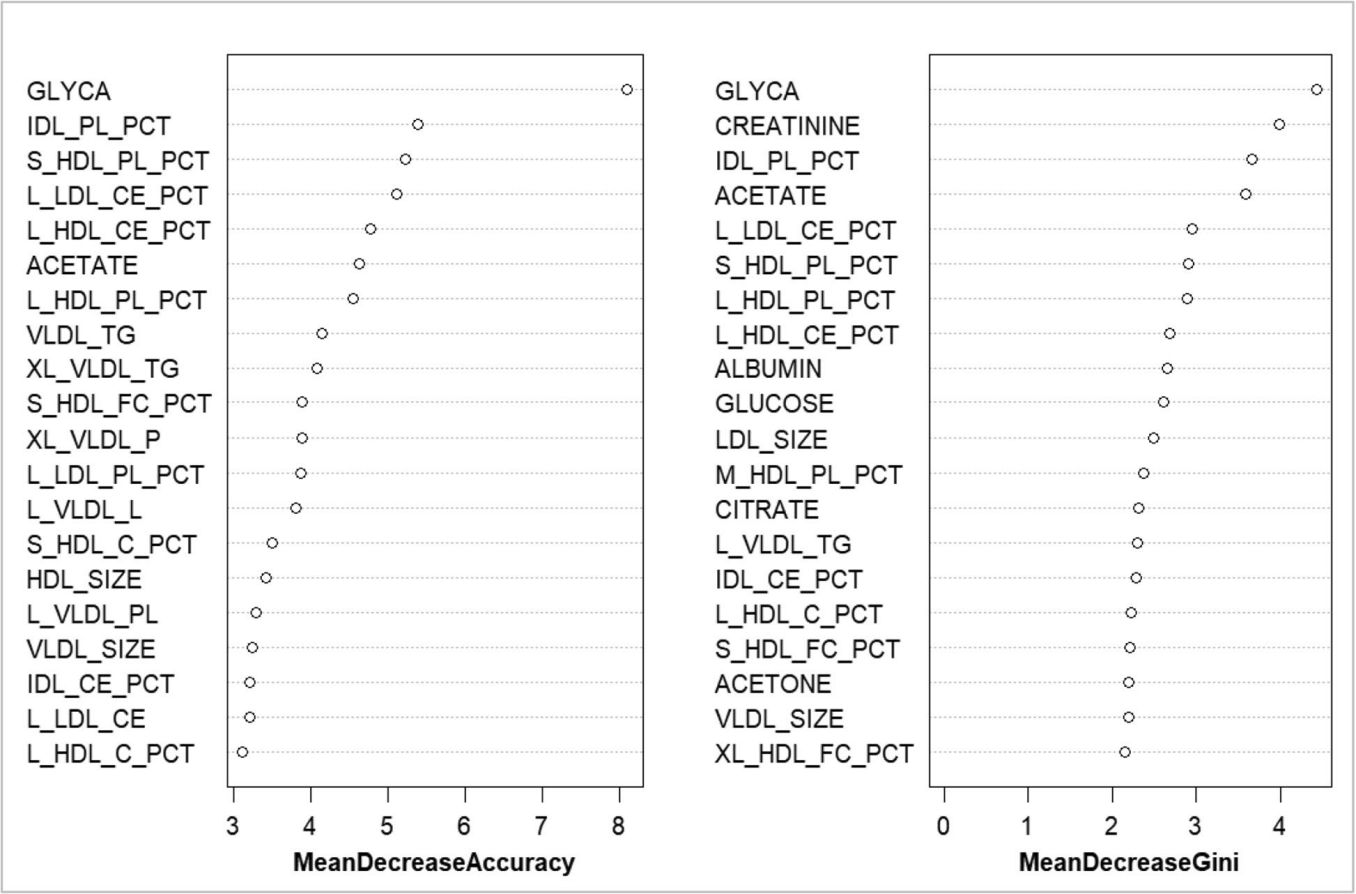
Variables screened using the two indicators based on the random forest model

This study used two indices to rank the importance of the 213 lipoproteins and metabolites. The top five important variables selected using the MDA indicator were glycoprotein acetylation (GLYCA), phospholipids to total lipids ratio in intermediate-density lipoproteins (IDL_PL_PCT), phospholipids to total lipids ratio in small high-density lipoproteins (S_HDL_PL_PCT), percentage of cholesteryl esters within low-density lipoprotein (L_LDL_CE_PCT), and cholesteryl esters to total lipids ratio in large HDL (L_HDL_CE_PCT). The top five most important variables selected using the MDG indicators were GLYCA, creatinine, IDL_PL_PCT, acetate, and L_LDL_CE_PCT. The top 20 variables screened using the two indicators are presented in Fig. 1 and Table 3.

**Table 3:** Variables screened using the two indicators based on the random forest model

Numbering	MeanDecreaseAccuracy		Numbering	MeanDecreaseGini	
	Variable	Value		Variable	Value
1	GLYCA	8.0929545	1	GLYCA	4.426083
2	IDL_PL_PCT	5.3943304	2	CREATININE	3.984054
3	S_HDL_PL_PCT	5.2229553	3	IDL_PL_PCT	3.662835
4	L_LDL_CE_PCT	5.1077540	4	ACETATE	3.587541
5	L_HDL_CE_PCT	4.7717346	5	L_LDL_CE_PCT	2.954548
6	ACETATE	4.6262435	6	S_HDL_PL_PCT	2.901989
7	L_HDL_PL_PCT	4.5528902	7	L_HDL_PL_PCT	2.885128
8	VLDL_TG	4.1495399	8	L_HDL_CE_PCT	2.679472
9	XL_VLDL_TG	4.0905157	9	ALBUMIN	2.653867
10	S_HDL_FC_PCT	3.8902676	10	GLUCOSE	2.611271
11	XL_VLDL_P	3.8866123	11	LDL_SIZE	2.487458
12	L_LDL_PL_PCT	3.8786212	12	M_HDL_PL_PCT	2.371273
13	L_VLDL_L	3.8077384	13	CITRATE	2.310676
14	S_HDL_C_PCT	3.4992093	14	L_VLDL_TG	2.291144
15	HDL_SIZE	3.4256874	15	IDL_CE_PCT	2.281446
16	L_VLDL_PL	3.2869513	16	L_HDL_C_PCT	2.217397
17	VLDL_SIZE	3.2508577	17	S_HDL_FC_PCT	2.206338
18	IDL_CE_PCT	3.2162258	18	ACETONE	2.200589
19	L_LDL_CE	3.2096993	19	VLDL_SIZE	2.188805
20	L_HDL_C_PCT	3.1233766	20	XL_HDL_FC_PCT	2.152936

### Variables screened using the Lasso regression model

**Fig. 2:**
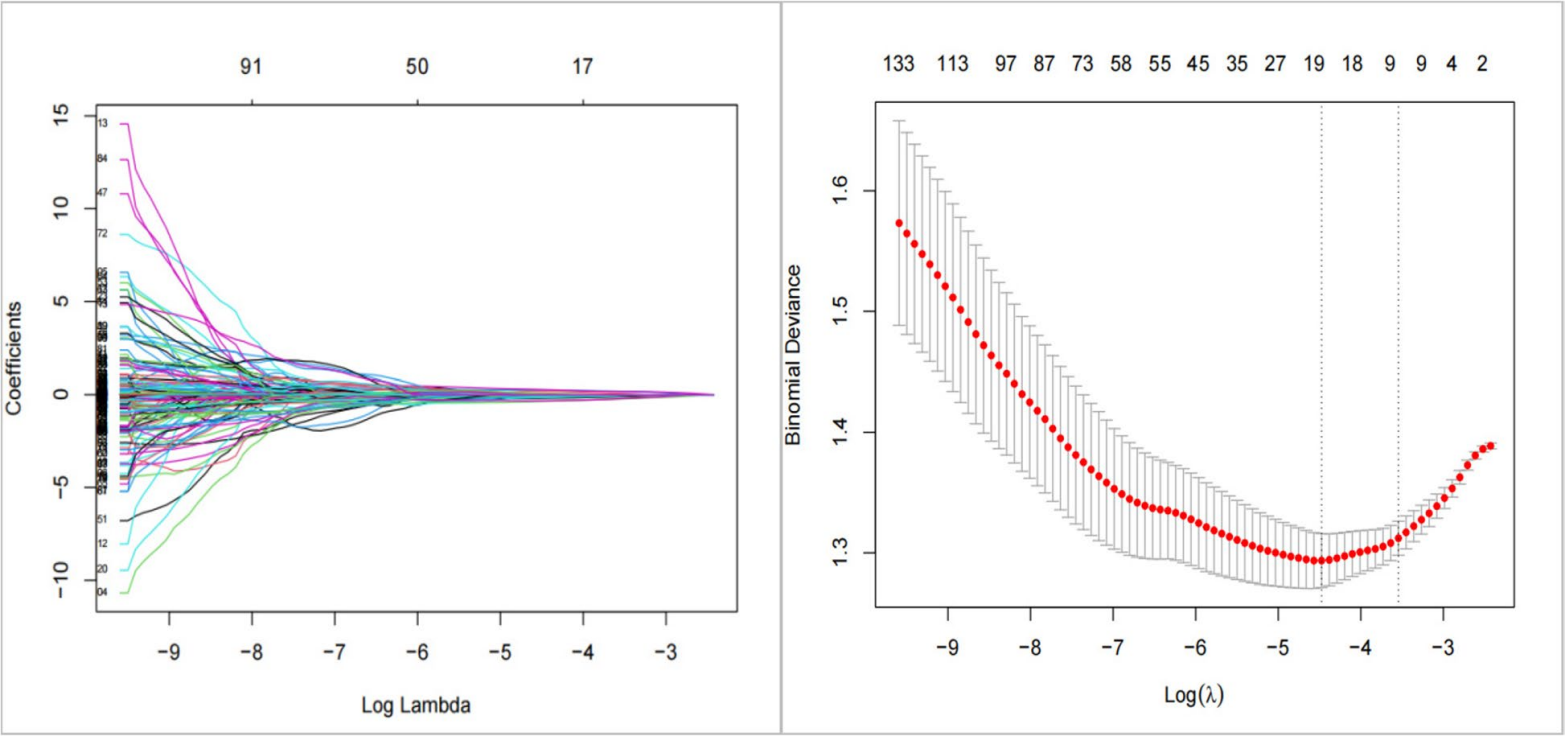
Top 19 variables screened using the Lasso regression model

The Lasso regression model was used to rank 213 lipid and metabolite variables. The coefficients of the variables with high importance were retained, whereas those with low importance were dismissed. In the final selection, 19 variables were retained (Fig. 2). The absolute values of the coefficients indicated the variables’ contributions to the model, with larger values signifying greater contributions. Among the retained variables, the top five with higher absolute coefficients were IDL_PL_PCT, L_HDL_PL_PCT, GLYCA, creatinine, and acetate. The ranking of the importance of these 19 variables is presented in Table 4.

**Table 4:** Top 19 variables screened using the Lasso regression model

Numbering	Variable	Coefficient	Numbering	Variable	Coefficient
1	IDL_PL_PCT	-0.35634199	11	XXL_VLDL_FC	-0.06525833
2	L_HDL_PL_PCT	-0.32669529	12	OMEGA_3	-0.06366583
3	GLYCA	0.27019005	13	PYRUVATE	-0.06112266
4	CREATININE	0.24233156	14	XL_HDL_TG_PCT	-0.05989037
5	ACETATE	-0.19706259	15	S_HDL_PL	-0.03986326
6	XL_VLDL_PL_PCT	0.17481366	16	L_VLDL_FC_PCT	0.03764699
7	M_VLDL_CE_PCT	-0.14568852	17	XXL_VLDL_PL	-0.03737671
8	ALBUMIN	-0.13129624	18	XL_HDL_PL_PCT	0.03595478
9	LDL_SIZE	-0.10019704	19	ACETONE	-0.02999795
10	GLUCOSE	0.07151750			

### Variables screened using the CatBoost algorithm

**Fig. 3:**
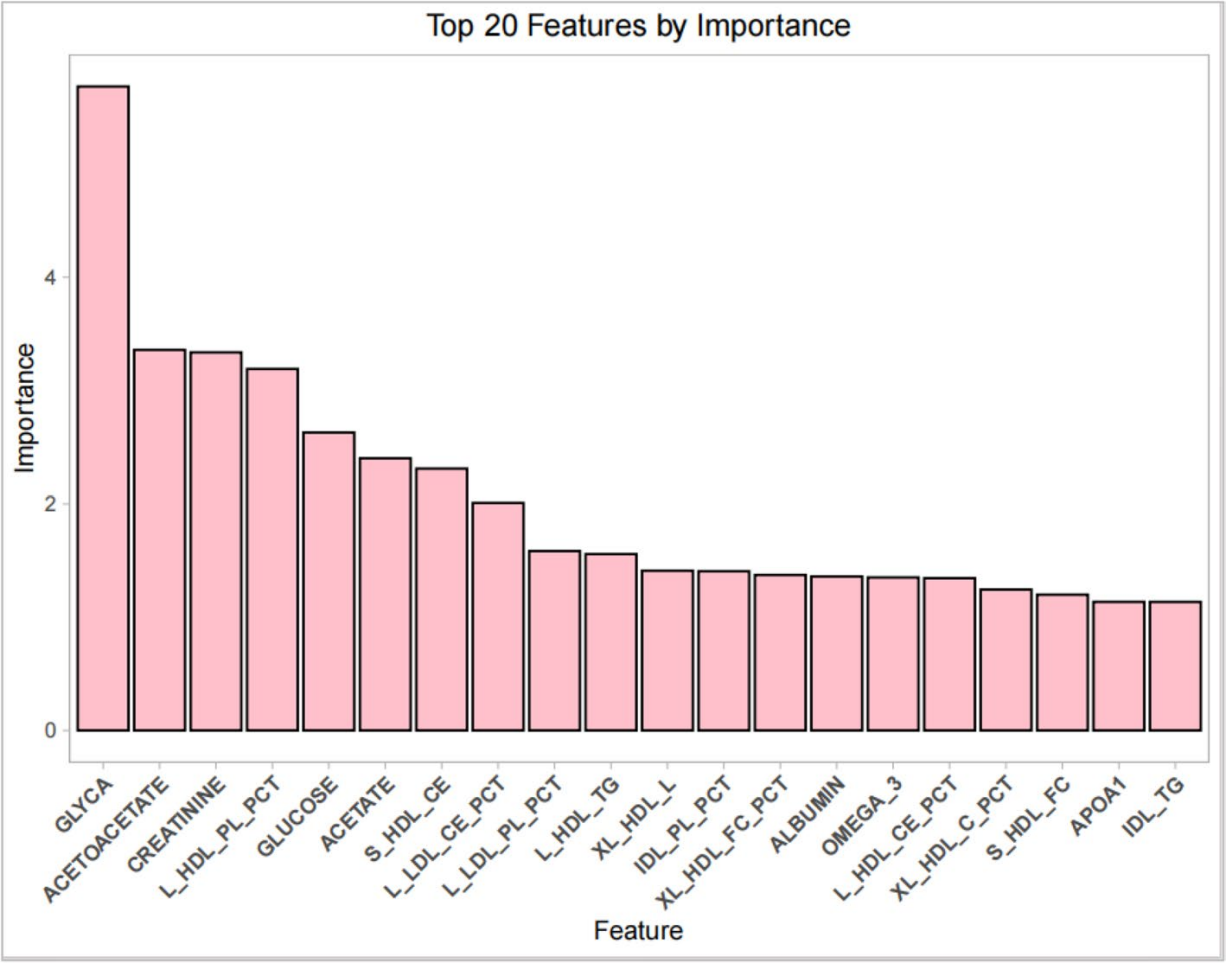
Top 20 variables screened using the CatBoost algorithm

The CatBoost algorithm was used to assess the importance of 213 lipid and metabolite variables. The magnitude of the gain was used to measure each variable’s contribution to the model performance, with higher gain values indicating greater contributions. The variables were ranked based on the magnitude of their gains. The top five variables were GLYCA, acetoacetate, creatinine, L_HDL_PL_PCT, and glucose. The top 20 variables screened using the CatBoost algorithm are presented in Fig. 3 and Table 5.

**Table 5:** Top 20 variables screened using the CatBoost algorithm

Numbering	Variable	Gain	Numbering	Variable	Gain
1	GLYCA	5.6847263	11	XL_HDL_L	1.4111951
2	ACETOACETATE	3.3583078	12	IDL_PL_PCT	1.4055307
3	CREATININE	3.3360474	13	XL_HDL_FC_PCT	1.3737200
4	L_HDL_PL_PCT	3.1894335	14	ALBUMIN	1.3603727
5	GLUCOSE	2.6285391	15	OMEGA_3	1.3515118
6	ACETATE	2.4005382	16	L_HDL_CE_PCT	1.3450783
7	S_HDL_CE	2.3091079	17	XL_HDL_C_PCT	1.2445356
8	L_LDL_CE_PCT	2.0090769	18	S_HDL_FC	1.1996838
9	L_LDL_PL_PCT	1.5845339	19	APOA1	1.1360386
10	L_HDL_TG	1.5572068	20	IDL_TG	1.1357228

### Random forest prediction model construction

**Fig. 4:**
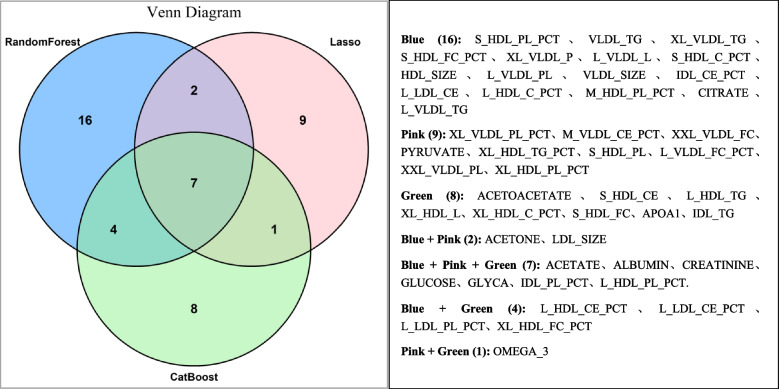
Venn diagram of the overlap of the selected variables using the three methods

Based on the random forest, LASSO regression, and CatBoost algorithms, 14 variables were selected: acetate, acetone, albumin, creatinine, glucose, GLYCA, IDL_PL_PCT, L_HDL_CE_PCT, L_HDL_PL_PCT, L_LDL_CE_PCT, L_LDL_PL_PCT, LDL_size, omega_3, and free cholesterol to total lipids ratio in very large HDL. The aforementioned 14 variables, along with age, sex, and marital status, were included in the random forest prediction model. Figure 4 depicts the 14 selected variables.

**Fig. 5:**
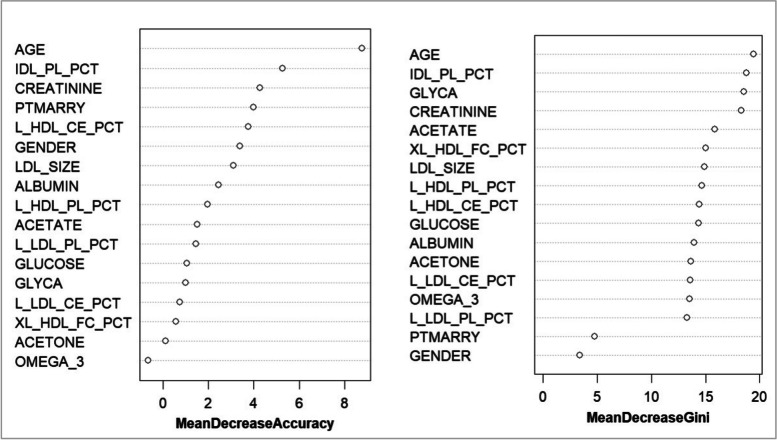
Variable importance ranking in the random forest prediction model based on the Mean Decrease Accuracy and Mean Decrease Gini

The random forest model achieved optimal results with “mtry” (number of features used in each tree) set to 3 and “ntree” (number of trees in the model) set to 300. The model exhibited an accuracy of 71.01%, a sensitivity of 79.59%, a specificity of 65.28%, and an AUC (95% CI) of 0.724 (0.645–0.804). The importance rankings of the variables in the random forest prediction model based on MDG and MDA showed some differences. The top five variables selected using the MDA were age, IDL_PL_PCT, creatinine, marital status, and L_HDL_CE_PCT. The top five variables selected using the MDG were age, IDL_PL_PCT, GLYCA, creatinine, and acetate. Notably, age, IDL_PL_PCT, and creatinine were among the top five in both rankings, underscoring their crucial roles in AD onset. The variable importance rankings in the random forest prediction model are presented in Fig. 5 and Table 6.

**Table 6:** Top 17 variables screened using the random forest model

Numbering	Mean Decrease Accuracy		Numbering	Mean Decrease Gini	
	Variable	Value		Variable	Value
1	AGE	8.751644698	1	AGE	19.43709257
2	IDL_PL_PCT	5.267551238	2	IDL_PL_PCT	18.75051635
3	CREATININE	4.272402103	3	GLYCA	18.51842448
4	PTMARRY	3.992261285	4	CREATININE	18.28195316
5	L_HDL_CE_PCT	3.754458302	5	ACETATE	15.87359581
6	GENDER	3.377002136	6	XL_HDL_FC_PCT	15.03763361
7	LDL_SIZE	3.119752591	7	LDL_SIZE	14.90034705
8	ALBUMIN	2.458896046	8	L_HDL_PL_PCT	14.64282445
9	L_HDL_PL_PCT	1.970092350	9	L_HDL_CE_PCT	14.40590370
10	ACETATE	1.518452241	10	GLUCOSE	14.36430505
11	L_LDL_PL_PCT	1.465085481	11	ALBUMIN	13.97586022
12	GLUCOSE	1.056593961	12	ACETONE	13.64832198
13	GLYCA	1.011491047	13	L_LDL_CE_PCT	13.61667028
14	L_LDL_CE_PCT	0.754445650	14	OMEGA_3	13.52545686
15	XL_HDL_FC_PCT	0.573652684	15	L_LDL_PL_PCT	13.32199892
16	ACETONE	0.115878414	16	PTMARRY	4.742327863
17	OMEGA_3	-0.636658927	17	GENDER	3.376408026

## Discussion

Recently, machine learning techniques have been applied in various fields. Medically, researchers have utilized machine learning to analyze extensive healthcare data [34, 35] for more accurate diagnoses and disease predictions. The random forest algorithm performs well in AD [36, 37], effectively enhancing the precision of predictive models. The uniqueness of the random forest algorithm lies in constructing each decision tree through random sampling, allowing it to model based on different subsets of data and features [37]. This helps mitigate the risk of overfitting, improves the predictive accuracy of the model, and ensures good generalization performance. Additionally, the random forest model can indicate the relative importance of variables and enhance the interpretation of the results.

Zhang et al. [38] conducted a study investigating the association between serum total cholesterol (TC) levels and neuropsychological performance, as well as intrinsic functional networks in non-demented older adults. They utilized ANCOVA analysis, adjusting for age, gender, and education years to compare neuropsychological performance between the two groups. The study found that in nondemented older adults, higher serum cholesterol levels were associated with disrupted functional connectivity in the salience network (SN). Proitsi et al. [39] conducted a study involving nontargeted lipidomics analysis of plasma samples from 148 patients with AD and 152 elderly controls. They utilized both univariate and multivariate analysis methods and found that blood lipids hold promise as potential biomarkers for AD. This discovery may lead to the emergence of new therapeutic strategies. In a study conducted by Chung et al. [40], they evaluated the relationship between LDL cholesterol (LDL-C) and gray-matter volume (GMV) in a community-based population without stroke or dementia. Using multiple linear regression analysis, they found that low circulatory LDL-C levels, in combination with hypertension, appeared to have a combined detrimental effect on posterior cingulate GMV, white matter hyperintensities (WMH), and verbal memory.

This study initially used the random forest, Lasso regression, and CatBoost algorithms to rank and filter the importance of the 213 lipid and metabolite variables included in this study. While determining the final modeling variables, the results of variable selection from using these three machine learning methods were comprehensively considered, which could reduce the probability of occasional variable selection using a single method and ensure the reliability of the modeling results. Among the top 20 variables selected using each method, seven variables, including acetate, albumin, creatinine, glucose, GLYCA, IDL_PL_PCT, and L_HDL_PL_PCT, were co-screened using all three methods, indicating the significant role of these variables in predicting AD onset. Finally, by employing the random forest model and generating a visualization of variable importance rankings, we visualized the importance ranking of the variables contributing to AD onset.

The results of the AD risk prediction model in this study indicated that the importance rankings of the variables age, IDL_PL_PCT, and creatinine, after being selected using the MDA and MDG indices, all ranked within the top five. This suggests that these three variables play crucial roles in AD onset. AD is one of the most common age-related neurodegenerative diseases [41], and its incidence significantly increases with age [42]. Removing triglycerides from very low-density lipoprotein in muscles and adipose tissues can lead to the formation of cholesterol-rich IDL particles, and these IDL particles can promote atherosclerosis [43]. The central nervous system is rich in lipids, primarily located on biological membranes, maintaining the structure and function of the central nervous system [16]. Changes in the lipid composition of the brain and plasma have been widely observed in patients with AD [44]. Phospholipids are key components of the cell membrane and can lead to synaptic dysfunction in patients with AD [45]. Physical activity and exercise prevent or delay AD onset [46], with lipid levels in patients with AD who exercise regularly lower than in those without AD [47]. Creatinine concentrations in the cerebrospinal fluid of patients with AD were significantly higher than in those without AD, which may be related to the overuse of creatine phosphate [48]. This result is consistent with the conclusions of this study. Creatinine is a byproduct of phosphocreatine [49], which stores high-energy phosphate bonds and releases energy when the glucose supply is insufficient. These results suggest that a significant increase in creatinine concentration in the cerebrospinal fluid of patients with AD causes an imbalance in their energy metabolism.

In conclusion, age, IDL_PL_PCT, and creatinine were the key factors identified that influence AD onset. Clinical screening and regular monitoring of lipoproteins and their metabolites in older patients can provide new perspectives for early AD diagnosis and prevention.

### Strengths and limitations

The strength of this study was that three machine learning methods were used for the preliminary screening of variables, which reduced the probability of chance in the screening of variables using a single method. Finally, the screening results of the three methods were combined for modeling, which improved the accuracy of the model to an extent.

However, this study had some limitations. First, this was a cross-sectional study; therefore, we only focused on the measurements of key lipoproteins and metabolites that affect AD pathogenesis at a certain point in time and did not consider the relationship between the longitudinal dynamic trajectories of these factors and AD pathogenesis. Second, no follow-up period was observed; therefore, investigating the impact of these key lipoproteins and metabolites on AD morbidity was impossible. Third, no standard range of health values for the studied variables was observed.

## Conclusion

This study identified age, IDL_PL_PCT, and creatinine as key factors closely associated with the onset of AD. Age is a well-established risk factor for AD, and this study further substantiates its significance in AD development. The discovery of IDL_PL_PCT provides novel insights into the relationship between lipid metabolism abnormalities and AD, offering researchers and clinicians potential avenues for investigation. The elevated concentration of creatinine in patients with AD suggests a potential link to energy metabolism imbalance. It can serve as an adjunct diagnostic marker, enhancing our understanding of AD pathogenesis and presenting potential therapeutic targets. For older individuals, closer monitoring and assessment should be carried out to facilitate early detection and increase the likelihood of diagnosing AD. Therefore, lipid metabolism management should be emphasized in patient care, including diet control, moderate exercise, and potential pharmacological interventions to maintain healthy lipid status.

## Data Availability

The datasets generated and analyzed during the current study are available in the [ADNI] database.

## Abbreviations

AD: Alzheimer’s disease
Aβ: β-Amyloid proteins
APP: Amyloid precursor protein
MAMs: mitochondria-associated endoplasmic reticulum membranes
CM: chylomicrons
VLDL: very-low-density lipoproteins
IDL: intermediate-density lipoproteins
LDL: low-density lipoproteins
HDL: high-density lipoproteins
ADNI: Alzheimer’s Disease Neuroimaging Initiative
GMV: gray-matter volume
IDL_PL_PCT: Phospholipids to total lipids ratio in intermediate-density lipoproteins
MDA: Mean Decrease Accuracy
MDG: Mean Decrease Gini
GLYCA: Glycoprotein acetylation
S_HDL_PL_PCT: Phospholipids to total lipids ratio in small high-density lipoproteins
L_LDL_CE_PCT: Percentage of cholesteryl esters within low-density lipoprotein
L_HDL_CE_PCT: Cholesteryl esters to total lipids ratio in large high-density lipoproteins
